# Technical Feasibility of TachoSil Application on Esophageal Anastomoses

**DOI:** 10.1155/2015/534080

**Published:** 2015-05-25

**Authors:** Leonie Haverkamp, Jelle P. Ruurda, Richard van Hillegersberg

**Affiliations:** Department of Surgery, University Medical Center Utrecht, Heidelberglaan 100, 3584 CX Utrecht, Netherlands

## Abstract

* Purpose*. Sealing esophageal anastomoses with a sealant patch (TachoSil) containing human fibrinogen and thrombin may improve mechanical strength. The aim was to evaluate the technical feasibility of the application of a sealant patch in upper gastrointestinal surgery.* Methods*. In total 15 patients, 18–80 years old, undergoing thoracolaparoscopic esophagectomy with esophagogastrostomy or laparoscopic total gastrectomy with esophagojejunostomy was included. Different techniques of anastomotic TachoSil patch application were tested and recorded on video.* Results*. TachoSil was successfully applied to the esophagogastrostomy (*n* = 11) and to the esophagojejunostomy (*n* = 4). A median of 2 (1–6) attempts was necessary to reach successful application. The median duration was 7 (3–26) minutes before successful application was accomplished. The best technique in esophagectomy was the application of TachoSil with the use of 2 cellophane sheets. For total gastrectomy, the patch was folded into a harmonica shape and wrapped around the esophagojejunostomy. Although not significant, the number of attempts and time to success showed a decreasing trend along with the increased experience.* Conclusion*. Application of TachoSil as a sealant of esophageal anastomoses was technically feasible. Future studies may investigate the value of TachoSil application on the prevention of anastomotic leakage.

## 1. Introduction 

Upper gastrointestinal cancer is surgically treated by means of esophagectomy or gastrectomy. A feared complication of these complex procedures is the postoperative development of anastomotic leakage. The incidence of leakage of the esophagogastrostomy after esophagectomy has been reported to be in the range of 5–26% in tertiary referral centers [1, 2]. The leakage rates of the esophagojejunostomy after total gastrectomy vary between 4 and 15% [3, 4]. Leakage may result in spill of gastrointestinal contents, followed by the development of fistula, wound infection, abscess, mediastinitis, empyema, and sepsis. This results in an extended postoperative course in the majority of patients due to prolonged hospital stay, admission to the intensive care unit, and reinterventions [5]. It is hypothesized that additional sealing of the anastomosis with a fibrin patch (TachoSil) containing a human fibrinogen and thrombin may improve mechanical strength and might potentially prevent anastomotic leakage. Its application on esophageal anastomoses in rats was associated with increased mechanical strength [6]. However, whether its additional application on human esophageal anastomoses is possible has not yet been investigated. The aim of this study was to evaluate the technical feasibility of the application of a sealant patch in esophageal surgery.

## 2. Materials and Methods

This trial was conducted in accordance with the World Medical Association Declaration of Helsinki. Approval for the study protocol by the Institutional Review Board of the University Medical Centre Utrecht was obtained. The recruitment period was from September 2012–November 2013. All patients were required to sign informed consent to enroll in the trial. Recruitment took place at the outpatient clinic at the Department of Surgery at the University Medical Centre Utrecht.

This single-center trial was designed to evaluate the technical feasibility of intraoperative additional TachoSil (TachoSil, Takeda, Zurich, Switzerland) application on conventional esophageal anastomoses. The investigational medicinal product was a TachoSil patch (9.5 × 4.8 cm), which consisted of an active yellow side with human thrombin (2.0 IU/cm^2^) and human fibrinogen (5.5 mg/cm^2^) and a supportive white side that consisted of collagen. The patch was applied intraoperatively after conventional anastomosis was performed. According to the study protocol, the patch had to cover at least 1-2 cm beyond the margins of the anastomotic line. The number of patches applied until success was determined by the treating surgeon with a maximum of 7 patches. In case multiple patches were used, the overlap between patches had to be 1-2 cm. In patients that underwent esophageal resection a hand-sewn end-to-side cervical esophagogastrostomy was constructed [7], after which TachoSil was applied. The patients that received laparoscopic total gastrectomy with jejunal pouch reconstruction had TachoSil applied after the mechanical end-to-end esophagojejunostomy.

The procedure of application was captured on video for further technical analysis. The primary efficacy parameter of the study was feasibility, which was defined on a 10-point action scale. The 10 items were scored (success or failure). A score of 10 out of 10 successful actions was regarded as an effective procedure. Before the study started it was determined that the application of TachoSil was regarded to be feasible if >12/15 patients (>80%) scored 10 out of 10 items on the 10-point action scale (Table 1). TachoSil is known to be degraded enzymatically in approximately 24 weeks after application. A follow-up of 9 months was completed in all patients to evaluate possible side effects such as benign anastomotic stenosis.

IBM SPSS Statistics version 20 (IBM Corp., Chicago, Illinois, USA) was used for the statistical analysis. For continuous variables the medians and ranges were calculated. For binominal variables, the proportions were calculated. The learning curve was analyzed by means of a linear regression analysis.

### 2.1. Technical Notes

Different techniques of anastomotic TachoSil patch application were tried out to compare the advantages and shortcomings of the methods of application. The cigarette role technique involved rolling the TachoSil patch into a cigarette shape and wrapping it around the anastomosis. The finger glove technique was used to separate the patch from the anastomotic tissue before placing it in the correct position. The cellophane sheet technique consisted of 2 cellophane sheets enclosing the patch. After proper placement the cellophane sheets were removed from the patch. The harmonica technique consisted of folding the patch into a harmonica shape to insert it into the abdominal cavity. Next, the patch was unfolded and wrapped around the anastomosis.

## 3. Results

A total of 20 patients undergoing an elective laparoscopic total gastrectomy with an esophagojejunostomy or laparoscopic esophagectomy with a planned esophagogastric anastomosis were included in the trial. Of these, 5 patients were excluded intraoperatively due to conversion of surgical procedure to either an esophagectomy and gastrectomy, with colonic interposition (*n* = 1), extended total gastrectomy with intrathoracic anastomosis (*n* = 3), or no resection due to peritoneal carcinomatosis (*n* = 1). This yielded 15 eligible patients for TachoSil application.

The 15 included patients consisted of 11 males and 4 females with an average age of 59 (24–80) (Table 1). Esophageal resection was performed in 11 patients, whereas total gastrectomy was performed in 4 patients. All patients (15/15) received a successful placement of the TachoSil patch. A median of 1 (1–6) attempt was necessary to reach successful application after esophagectomy and 2 (1–3) after gastrectomy (Table 2). The total duration was 5 (3–26) minutes before successful application was accomplished after esophagectomy and 7 (5–18) minutes after gastrectomy. Anastomotic leakage requiring reoperation occurred in 2 patients. Both showed necrosis of the gastric conduit, which was drained after which they subsequently recovered. Postoperative benign anastomotic stenosis was seen in 5 patients.

### 3.1. Esophagectomy—Cervical Esophagogastrostomy

The first technique that was evaluated was the so-called cigarette technique, which was performed in 3 patients. The TachoSil patch was rolled to cigarette shape and pulled at the dorsal side of the fully completed esophageal anastomosis. Next, the patch was unrolled and wrapped around the ventral side of the anastomosis as well, so that both ends of patch overlapped. Finally, pressure was applied to the patch. The difficulty that was experienced with this technique was the rupture of the patch. This resulted in laceration of the patch or increased cohesiveness, causing a sticky structure, which was difficult to stretch and maneuver. To prevent this cohesion, the patch was placed in a cut open finger of a surgical glove before placement at the dorsal side of the anastomosis. This technique was also performed in 3 patients. The challenge of this technique was in removing the glove. Due to its elasticity and open shape, the patch attracted fluid, resulting in increased adhesive properties.

**Figure 1:**
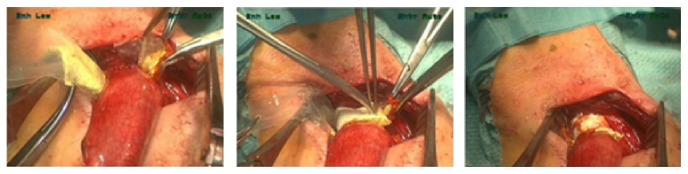
Strengthening the esophagogastric anastomosis with a TachoSil patch. Application by means of 2 cellophane sheets.

The final strategy to overcome these difficulties was to place the patch in between 2 cellophane sheets that were 1 cm larger than the actual patch (Figure 1). Lubricating gel was applied to the outside of the sheets to facilitate their removal after placement of the patch. The patch, enclosed by the sheets, was grasped with a clamp that was located at the dorsal side of the anastomosis. Next, the clamp was pulled from the right lateral side of the anastomosis to the left, covering the entire dorsal side of the anastomosis. The 2 cellophane sheets were removed by pulling them one by one to the left lateral side. The ventral side of the anastomosis was covered, ensuring a 1-2 cm overlap between both ends of the patches. Pressure was applied to the TachoSil patch to ensure its adhesion to the anastomotic tissue. This strategy turned out to be the most favorable, since it was successfully performed in 1 attempt in 5/5 patients. The learning curve for the 11 patients undergoing esophagectomy showed a trend towards a decrease in number of attempts necessary before successful application over time (*P* = 0.059). The shortening of duration of the patch application was not significant over time (*P* = 0.196).

### 3.2. Timing of Application

For the first 2 patients, the patch was applied after the entire anastomosis was completed. However, the limited available space that remained, hampered the positioning of the patch. Therefore, in the next 2 patients the application of the TachoSil patch was applied directly after the dorsal side of the anastomosis was completed (Table 2). After TachoSil placement, the ventral side of the anastomosis was sutured. In another patient the patch was placed at the area where the anastomosis was going to be constructed. The actual placement was easy in this case, but the construction of the anastomosis was hindered by the patch. The favored timing of patch placement was directly after the dorsal side of the anastomosis was completed, before suturing the ventral side of the anastomosis.

### 3.3. Total Gastrectomy—Esophagojejunostomy

**Figure 2:**
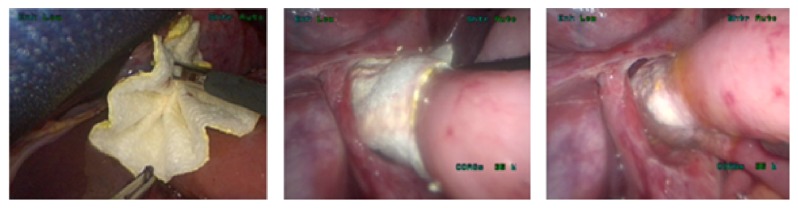
Strengthening the esophagojejunostomy with a TachoSil patch. Application by means of folding the patch in a harmonica shape.

In all total gastrectomy patients (*n* = 4) the conventional esophagojejunostomy was stapled before a TachoSil patch was applied. The TachoSil patch was folded in a harmonica shape extracorporeally (Figure 2). Next, it was inserted through a Dextrus port (Ethicon Cincinnati, OH, USA) and positioned ventrally to the anastomosis with the active side directed at the anastomosis. Next the harmonica shape was unfolded by gently pulling either side of the patch. The patch was slowly advanced over the anastomosis and wrapped around it. To ensure adhesion, pressure was applied to the patch. No learning curve was found for TachSil application in these patients.

## 4. Discussion

The objective of this study was to evaluate the technical feasibility of the application of a sealant patch in esophageal surgery. This study has shown that the application of TachoSil on esophageal anastomoses is technically feasible. The preferred method of TachoSil application on the cervical esophagogastrostomy was by means of 2 cellophane sheets. For the esophagojejunostomy after total gastrectomy the harmonica technique was favored.

Patients undergoing either an esophagogastrostomy or esophagojejunostomy were included in this study to allow for the development of specific application techniques for both anastomoses. In preparation of this study, preliminary investigations were performed to evaluate the possibilities of TachoSil application on the esophagus in an in vivo rat model and in human cadavers [6]. The animal study was designed to test the anastomotic strength after TachoSil application. This was measured by means of testing the burst pressure of the anastomosis. Therefore, the rats were sacrificed on postoperative *d* 0, 3, 5, and 7 before anastomotic leakage developed. The anastomotic strength was improved after TachoSil application. In the human cadaver pilot we found that sufficient space was available to place the patch onto the anastomosis of the human cadaver. However, in live patients the space at the dorsal side of the anastomosis in the neck turned out to be limited, which resulted in the manipulation of the anastomosis during the application of the patch.

Different techniques of anastomotic TachoSil patch application were tried out to compare the advantages and shortcomings of the methods of application. The aim was to gradually develop the best technique to apply the patch. The main difficulty with the cigarette technique was the laceration of the TachoSil patch in case of substantial pulling force on the patch. With regard to the finger glove technique, the removal of glove was hampered, due to the elasticity of the material. Moreover, we experienced that in both techniques the patch could get in contact with fluids, leading to increased cohesiveness when force was applied to maneuver it. These problems were solved with the use of 2 lubricated cellophane sheets enclosing the TachoSil patch. Successful application was achieved in 1 attempt in 5/5 patients.

Patients receiving a laparoscopic total gastrectomy with subtotal esophagectomy and intrathoracic anastomosis (*n* = 3) were excluded from the trial, since the intrathoracic cavity limits visibility and maneuverability when accessed transhiatally. Providing gentle application of the TachoSil patch by experienced laparoscopic surgeons, no difficulties were encountered in the laparoscopic total gastrectomy group.

Thus far, no other studies evaluating TachoSil application on esophageal anastomoses have been performed. Interestingly, its successful use in closure of pharyngocutaneous fistulas in 3 patients with laryngeal or oropharyngeal cancer has been described [8]. The safety and efficacy of TachoSil application have been demonstrated in larger series of patients undergoing pulmonary, liver, and gynecologic surgery [9–11]. Also, TachoSil application in colorectal anastomoses was found to be feasible and well tolerated [12].

As an alternative method to reinforce the anastomosis, an omental wrap has been used in gastric bypass surgery and in colorectal anastomosis [13, 14]. Also, a recent study evaluated the use of a pedicled omentum in esophagogastric anastomoses [15]. This prospective RCT showed a significant decrease in leak rate in the group of patients with a reinforced anastomosis by means of an omental wrap.

The objective of the present study was to evaluate the technical feasibility of the application of a sealant patch in esophageal surgery. We designed this study to develop the optimal technique to apply a sealant patch onto an esophageal anastomosis. As a result, data on leakage are not valid based on this small scale study. Thus, the finding that we encountered, 2 anastomotic leakages requiring reoperation and 5 postoperative benign anastomotic stenoses, is of limited value. With increasing experience in the application of the TachoSil patch, the rate of anastomotic leakage may be evaluated. An accurately powered randomized controlled trial could lead to reliable results with regard to leakage rate. In addition, the occurrence of benign cervical stricture formation should be evaluated. The incidence of a benign postoperative stenosis ranges between 27 and 42% of all esophagectomies and leads to a reduction in quality of life [16–18]. After gastrectomy benign anastomotic structures are seen in up to 36% of patients [19]. This complication could be prevented by reducing leakage rates. On the other hand, a possible disadvantage of the application of a TachoSil patch might be the development of an anastomotic stenosis. It could be the case that the TachoSil patch reinforces the anastomosis too much, causing increased fibrosis. This should be evaluated in an accurately powered randomized controlled trial as well.

## 5. Conclusions

This study showed our initial experience with intraoperative topical application of TachoSil on esophageal anastomoses. It was demonstrated that this application was technically feasible on both esophagojejunostomy and esophagogastrostomy. Along with the increased experience, the number of attempts and time to success decreased. However, this difference was not significant in this small series of 15 patients. Future studies should evaluate the influence of reinforcement of the esophageal anastomosis on anastomotic leakage and development of benign postoperative stenosis, preferably in a randomized controlled trial.

## Figures and Tables

**Table 1:** Baseline characteristics of patients that received TachoSil.

	TachoSil application *N* = 15
Age^*∗*^	59 (24–80)
Gender (M : F)	11 : 4
BMI^*∗*^	23.8 (16.6–33.6)
Esophageal resection	11
Gastric resection	4
Gastroesophageal cancer	12
Benign disease	3
History of smoking	11
Comorbidities	
Pulmonary disease	4
Cardiac disease	5
Diabetes mellitus	3
Neoadjuvant chemoradiotherapy	7

^*∗*^Median (minimum–maximum).

**Table 2:** Evaluation of methods of application.

Patient	Surgery	Method of application	Attempts	Duration application (min)	Timing of application
1	Esophagectomy	Cigarette roll	2	4	Entire anastomosis completed
2	Esophagectomy	Cigarette roll	6	26	Entire anastomosis completed
3	Esophagectomy	Cigarette roll	2	11	Dorsal side of anastomosis completed
4	Esophagectomy	Finger glove	1	5	Dorsal side of anastomosis completed
5	Gastrectomy	Harmonica shape	3	18	Entire anastomosis completed
6	Gastrectomy	Harmonica shape	1	5	Entire anastomosis completed
7	Esophagectomy	Finger glove	3	25	Entire anastomosis completed
8	Esophagectomy	Finger glove	3	17	Entire anastomosis completed
9	Esophagectomy	Cellophane	1	4	Dorsal side of anastomosis completed
10	Esophagectomy	Cellophane	1	5	Before starting handsewn sutures
11	Esophagectomy	Cellophane	1	3	Dorsal side of anastomosis completed
12	Esophagectomy	Cellophane	1	7	Dorsal side of anastomosis completed
13	Esophagectomy	Cellophane	1	4	Dorsal side of anastomosis completed
14	Gastrectomy	Harmonica shape	2	7	Entire anastomosis completed
15	Gastrectomy	Harmonica shape	2	8	Entire anastomosis completed

**Table 3:** Time-action score form for the harmonica technique of TachoSil application on esophagojejunostomy after laparoscopic total gastrectomy.

Action	Effectiveness	Number of actions (*n*)	Duration (s)
Folding TachoSil patch into harmonica shape	Success/failure		
Grasping TachoSil patch	Success/failure		
Inserting surgical instrument with TachoSil patch through trocar	Success/failure		
Moving TachoSil patch to location of anastomosis	Success/failure		
Turning TachoSil patch 180 (yellow side at the bottom)	Success/failure		
Placing TachoSil at anastomotic tissue	Success/failure		
Unfolding TachoSil patch	Success/failure		
Applying pressure at TachoSil patch	Success/failure		
Leaving TachoSil at anastomotic site	Success/failure		
Adherence of complete TachoSil patch	Success/failure		

## Appendix

See Table 3.
